# Sphingolipids and DHA Improve Cognitive Deficits in Aged Beagle Dogs

**DOI:** 10.3389/fvets.2022.646451

**Published:** 2022-07-13

**Authors:** Joseph A. Araujo, Sergi Segarra, Jessica Mendes, Andrea Paradis, Melissa Brooks, Sandy Thevarkunnel, Norton W. Milgram

**Affiliations:** ^1^InterVivo Solutions Inc., Fergus, ON, Canada; ^2^R&D Bioiberica S.A.U., Esplugues de Llobregat, Barcelona, Spain; ^3^CanCog Inc., Fergus, ON, Canada

**Keywords:** aging, cognition, executive function, canine, neurodegeneration, cognitive dysfunction syndrome

## Abstract

Canine cognitive dysfunction syndrome (CDS) is a disorder found in senior dogs that is typically defined by the development of specific behavioral signs which are attributed to pathological brain aging and no other medical causes. One way of objectively characterizing CDS is with the use of validated neuropsychological test batteries in aged Beagle dogs, which are a natural model of this condition. This study used a series of neuropsychological tests to evaluate the effectiveness of supplementation with a novel lipid extract containing porcine brain-derived sphingolipids (Biosfeen®) and docosahexaenoic acid (DHA) for attenuating cognitive deficits in aged Beagles. Two groups (*n* = 12), balanced for baseline cognitive test performance, received a daily oral dose of either test supplement, or placebo over a 6-month treatment phase. Cognitive function was evaluated using the following tasks: delayed non-matching to position (DNMP), selective attention, discrimination learning retention, discrimination reversal learning, and spatial discrimination acquisition and reversal learning. The effect of the supplement on brain metabolism using magnetic resonance spectroscopy (MRS) was also examined. A significant decline (*p* = 0.02) in DNMP performance was seen in placebo-treated dogs, but not in dogs receiving the supplement, suggesting attenuation of working memory performance decline. Compared to placebo, the supplemented group also demonstrated significantly improved (*p* = 0.01) performance on the most difficult pattern of the spatial discrimination task and on reversal learning of the same pattern (*p* = 0.01), potentially reflecting improved spatial recognition and executive function, respectively. MRS revealed a significant increase (*p* = 0.048) in frontal lobe glutamate and glutamine in the treatment group compared to placebo, indicating a physiological change which may be attributed to the supplement. Decreased levels of glutamate and glutamine have been correlated with cognitive decline, suggesting the observed increase in these metabolites might be linked to the positive cognitive effects found in the present study. Results of this study suggest the novel lipid extract may be beneficial for counteracting age-dependent deficits in Beagle dogs and supports further investigation into its use for treatment of CDS. Additionally, due to parallels between canine and human aging, these results might also have applicability for the use of the supplement in human cognitive health.

## Introduction

Aged dogs develop both neuropathology and cognitive decline paralleling several aspects of Alzheimer's disease that likely contribute to the behavioral signs associated with canine cognitive dysfunction syndrome (CDS) (1–8). Some of the neuropathological similarities include reduced brain volume, neuronal loss, impaired neurogenesis, and amyloid beta deposition (9), the latter of which has been shown to positively correlate with level of cognitive impairment (10). Behavioral changes characterizing CDS include alterations in activity, house soiling, recognition of family members and conspecifics, as well as disorientation and anxiety. Dogs also show neuropsychological evidence of cognitive impairment that includes age-related deficits in learning, memory, and executive function/attention which can serve as markers of neurodegeneration (1–4, 11, 12). Specifically, impairments in acquisition and in performance of a delayed non-matching to position (DNMP) task, which assesses working memory, are seen as early as middle age in dogs and DNMP performance can decline over months, independent of performance level (3, 4). Similarly, reversal learning performance and selective attention are impaired in old dogs compared to young, which reflect deficits in executive function and inhibitory attentional processes (13, 14). Both frontal cortex amyloid deposition and brain atrophy are negatively correlated with performance on the DNMP and reversal learning tasks, suggesting that frontal cortex neuropathology is linked to cognitive deficits in these domains (15, 16). In pet dogs, the sequalae of these neurodegenerative processes can result in behavioral changes that may lead to a diagnosis of CDS (1–6, 17). Importantly, we have previously demonstrated that age-related cognitive decline in dogs precedes behavioral changes such as alterations in sleep-wake patterns, interaction with humans, and environmental attention (1, 3).

Aged laboratory Beagles with naturally occurring cognitive decline and associated brain pathology provide a model for assessing potential cognitive benefits of pharmaceutical and nutritional interventions for CDS which may have translational value for Alzheimer's disease research (2, 6–8). Previously, we have shown that long-term treatment with nutritional interventions can augment cognition and/or modify pathological biomarkers of disease. For example, feeding of a canine diet enriched with antioxidants and mitochondrial cofactors improved performance across multiple cognitive domains as well as attenuated the cerebral deposition of amyloid-beta (18–22). Similarly, supplementation with medium chain triglycerides resulted in improved cognitive measures as well as improved mitochondrial function in aged dogs (23, 24) and a commercially available canine diet enhanced with medium chain triglycerides improved behavioral signs of CDS in pet dogs (25). Although similar supplements, including medium chain triglycerides, omega-3 fatty acids, and docosahexaenoic acid (DHA) have not been shown to improve human cognition to this extent (26–28), standardized canine diets may lack key nutrients, or the variety of nutrients, found in the human diet. As such, the effects of dietary supplementation in dogs may result in more robust effects and could therefore provide an explanation as to why the canine model of age-related cognitive impairment has not demonstrated the ability to predict success of nutritional interventions in human patients. In addition, the limited translational ability may also reflect different neuropsychological assessment tools compared to those used in human studies.

In the current study, we evaluated the effects of a novel combination of porcine brain-derived sphingolipids (described below) and DHA on cognitive measures in aged Beagle dogs. DHA is the most abundant polyunsaturated fatty acid in the brain and is involved in several processes including neuroprotection, regulation of neuroinflammation, synaptogenesis, and synaptic membrane function, among others (29, 30). When DHA enters the brain, it is integrated into membrane phospholipids and is re-released during neurotransmission as well as following brain injury (30). DHA is thought to have a protective effect against cognitive decline by limiting the production and accumulation of the amyloid beta peptide toxin and suppressing signal transduction pathways induced by amyloid beta, including those associated with neurofibrillary tangle pathology (31). Given the wide range of processes DHA supports, it is not surprising that a deficiency in this fatty acid can lead to a variety of neurodegenerative disorders (29, 32). Several studies have demonstrated cognitive benefits of increased DHA exposure in dogs as well as humans and other mammalian species (19, 33–35). In aged dogs, DHA supplementation improves visual processing measured using a contrast discrimination task when compared to a diet absent in DHA (36). In puppies, DHA dose dependently improves performance on cognitive tasks evaluating executive function, conditional discrimination learning, contrast sensitivity, and motor performance as well as improves retinal function assessed by electroretinography (37).

Sphingolipids are structural components of cellular membranes that are also involved in cell signal transduction and cell recognition. They are abundant in the central nervous system and are essential for its function. Alterations in sphingolipid metabolism can lead to rearrangement of cell membranes and the development of central nervous system deficits including cognitive and motor impairment and are found early in the course of Alzheimer's disease (38–41). Recent research developments propose the mechanisms that regulate cognition are lipid based, with sphingolipids playing an important role in learning and memory (42). Preliminary research of the specific sphingolipid formula evaluated here (i.e., Biosfeen®) suggested a reduction in oxidative stress and improved cognition in murine models of both Alzheimer's disease and accelerated aging (43, 44). We are not aware of studies evaluating the cognitive effects of sphingolipids in combination with DHA in dogs; however, in a previously conducted study evaluating a combination of DHA and pig-derived phospholipids, which included sphingomyelin, we found the supplement to improve working memory performance and quality of life in aged dogs (19). Therefore, we hypothesized that the combination of DHA and brain-derived sphingolipids from pigs would improve cognitive function and measures of brain health in aged dogs. Specifically, a subset of cognitive tasks that are sensitive to both canine aging and therapeutic intervention, as well as magnetic resonance spectroscopy for evaluation of brain metabolism, was used to assess the effectiveness of the supplement for improving or attenuating cognitive deficits and *in vivo* imaging parameters associated with brain aging in aged Beagle dogs.

## Materials and Methods

### Experimental Product

The experimental supplement was a combination of pig brain-derived sphingolipids, Biosfeen®, and DHA provided by Bioiberica, S.A.U. (Esplugues de Llobregat, Spain). The pig was chosen as the source of sphingolipids for this product due to the abundance of these molecules in the porcine brain. Biosfeen® was selected for evaluation in the current study based on preliminary findings of improved cognition in murine models of Alzheimer's disease and accelerated aging (43, 44). These studies also informed dose levels for the current study.

The supplement was provided in soft gelatin capsules containing 250 mg pork brain sphingolipids, 225 mg DHA and 90 mg eicosapentaenoic acid. The pork brain component of the extract consisted of approximately: 30% gangliosides, 35% sphingomyelins, and 10% ceramides, with other non-specified phospholipids and neutral lipids comprising the remaining lipid composition. Both the supplement and vehicle control (placebo) were prepared in identical gelatin capsules to ensure blinding during oral administration. Following baseline testing, subjects received a once daily administration (3 capsules) of their assigned treatment for the remainder of the study, which included 86 days of wash-in prior to 82 days of cognitive testing. Capsules were administered 1 h prior to cognitive testing procedures (±15 min).

### Subjects

Twenty-nine Beagle dogs of both sexes, maintained in the InterVivo Solutions Inc. colony, were cognitively screened for inclusion in the study at baseline; twenty-four were selected onto the treatment phase. One animal was excluded for declining health and the remaining 4 were excluded for committing the greatest number of non-responses during baseline testing.

The selected subjects ranged from 6.5 to 13.9 years in age (μ ± S.D. = 8.6 ± 2.52) at the start of the study. Based on previous findings that impairments in acquisition and in performance on the DNMP are seen as early as middle age (3, 4), a large age range was included in the present study to avoid limitations in detecting a treatment effect in a group that was too early or too late in the cognitive aging process. There was 1 male and 23 females included. The over-representation of females was due to the high availability of aged female retired breeders compared to retired male breeders; however, we previously have not found sex differences in cognitive performance of the tasks utilized here.

Dogs were individually fed to maintain body condition using a commercially available diet: Purina® ProPlan® Savor® Adult - Chicken and Rice Formula. Feed was offered once daily at the end of the day following completion of all test procedures. Water was provided *ad libitum*. Up to four dogs were group housed in pens measuring 1.5 x 4.9 m, which included raised resting platforms and a rotation of toys for enrichment. A combination of natural and fluorescent lighting was provided for the animals, resulting in a minimum of 12 h of light (and <12 h of dark if the day was longer).

### Study Design

A parallel group design was used to evaluate the effects of the novel lipid supplement compared to a placebo control. Technical staff collecting cognitive and observational data were blinded to treatment assignments. Initially, dogs were evaluated at baseline for performance on the DNMP, discrimination learning, and selective attention tasks (as described below). Suitable subjects were ranked in descending order on each of the baseline cognitive tests according to total number of errors. The sum of ranks was then calculated and used to allocate subjects into equivalent treatment groups based on performance and a two-tailed *t*-test was conducted to confirm an absence of group differences on any of the tests or at either delay within the DNMP task. Groups were chosen to be balanced for cognitive performance rather than age as aging is a heterogenous process with differing trajectories of decline occurring across individual subjects.

A group size of *n* = 12 was selected based on previous power analysis of historical DNMP data, which indicated groups of 12–14 dogs would result in at least 80% power and 10% effect size. Each group was assigned to a treatment condition (i.e., test or control) by drawing of lots. Subjects then received their respective treatment for the duration of the study (see Table 1). Following an 86-day wash-in, dogs were tested on the following tasks: spatial discrimination learning, spatial discrimination reversal, discrimination learning (retention from baseline), selective attention, discrimination reversal, and DNMP. As DNMP performance in middle aged dogs has been shown to worsen over time (3, 4), a wash-in period of approximately 3 months was chosen with the goal of observing treatment related attenuation of decline in DNMP performance.

**Table 1:** Study schedule.

**Phase**	**Study Day**	**Procedures**
Baseline	−57 to −48	DNMP Testing
	−47 to −33	Discrimination Learning Attention Testing^a^
	−32 to −30	Subject Selection & Group Allocation
	−13 to −2	Brain Imaging Procedures
Treatment	0 to 85	Beginning of Daily Dosing (Wash-In)
	86 to 126	Spatial Discrimination Learning and Reversal Testing
	129 to 133	Discrimination Retention Testing
	134 to 137	Attention Testing
	138 to 158	Discrimination Reversal Testing
	159 to 168	DNMP Testing
	176	Brain Imaging Procedures

a *Attention testing was initiated the day following completion of discrimination learning for each animal (i.e., when learning criteria was reached)*.

The first task performed during the treatment phase (i.e., spatial discrimination) was not performed at baseline. The rationale behind this was 2-fold. First, it would have added an additional layer of complexity when balancing groups for learning ability and therefore, we used the basic discrimination task to assess learning ability at baseline; secondly, since it is only possible to assess initial learning of a task once, we wanted to evaluate initial learning of the spatial discrimination task by treatment condition. Due to the absence of group differences in cognitive ability at baseline, this would increase our ability to identify treatment effects; however, the absence of baseline spatial discrimination testing precludes us from concluding that the groups were balanced at baseline for performance on this task.

### Apparatus and General Cognitive Test Procedures

The test apparatus, described previously (18, 45), consisted of a box measuring approximately 0.57 x 1.20 x 0.7 m. After entering the apparatus using a ramp, the dog was able to access the test stimuli and food rewards through adjustable openings at the front of the box using only its head. The test stimuli were presented to the dog on a sliding plastic tray that contained either three or four food wells. The test stimuli were placed on coasters and presented over the appropriate food wells and, after displacing the correct stimulus, the dog was allowed to retrieve the food in the well below. Dogs were allowed to correct their first incorrect response on each test session. All coasters were baited with large amounts of unobtainable food to prevent the dog from solving the tasks using olfactory cues. A partition with a hinged door was used to prevent the dog from observing the placement of test stimuli and rewards between trials or during the delays, as applicable. The tester lowered the hinged door and presented the tray to the dog for 3s before further advancing the tray and allowing the animal to respond. An inter-trial interval of 30 seconds was used for each task. A dedicated computer program (VariCog, Vivocore Inc., Toronto, ON) that controlled randomization procedures within a session and allowed the tester to record response latencies and subject responses was used for all cognitive tasks.

### Delayed Non-matching to Position Task

The DNMP task was conducted over 10 days during baseline and again for 10 days following 159 days of treatment to assess spatial short-term working memory (1, 4). Briefly, subjects were initially presented with a single object (i.e., white block) on the sliding tray. The block was positioned over one of 3 possible food-well locations and the dog was required to displace the block with its nose to uncover a food reward. The tray was then removed from the dog's sight and a delay was initiated. Following the delay, the subject was presented with two white blocks – one in the original location and one in a new location (1–4). The dog was required to select the block in the new (non-matching) location to obtain a reward. On each test day, subjects participated in a single session, with delays of 20 and 90 s divided equally among 12 trials. Percent correct responses at each delay served as the dependent variable for statistical analyses.

### Discrimination Learning and Reversal Task

The discrimination and reversal learning tasks assess learning ability and executive function, respectively (2, 4, 13, 14). The discrimination learning task was conducted at baseline and again following 129 days of treatment to assess learning retention from baseline. The reversal learning task was then conducted following 138 days of treatment. For the discrimination learning task conducted at baseline, dogs initially participated in a 10-trial preference test in which two objects differing in size, shape, and color (i.e., green block and yellow banana) were presented equally over the lateral food wells of the sliding tray, both of which were rewarded. The object selected most frequently by each dog, the dog's preferred object, was used as the rewarded stimulus for the subsequent learning sessions during which selection of the preferred object resulted in obtaining a food reward. Discrimination learning was conducted using 20 trials per day until a 2-stage learning criterion was reached or a maximum of 10 sessions. The first stage required subjects to achieve 90% correct responses, or greater, on one session or a minimum of 80% correct responses over two consecutive sessions. The second stage required subjects to achieve 70% correct responses, or greater, over two consecutive sessions immediately following passing of the first criterion. Retention testing was performed over 5 consecutive days, regardless of score. The rewarded object remained the same as baseline. Discrimination reversal testing was performed using the same 2-stage criterion or a maximum of 21 days. During reversal testing, the non-preferred object was the rewarded object. Cumulative errors for each subject on discrimination learning, discrimination retention, and reversal learning served as the dependent variable for statistical analyses.

### Selective Attention Task

The selective attention task was identical to discrimination learning; however, the preferred object was presented with either 0, 1, 2, or 3 negative stimuli (the non-preferred object) to serve as distractors (4). The number of distractors was divided equally and presented randomly over 20 trials in each session. This task was used to test attention over 4 days at baseline, following completion of discrimination learning, and again for 4 days following 134 days of treatment, i.e., immediately following the learning retention sessions. On each trial, the subject was required to respond to the preferred object to obtain the food reward and cumulative errors at each distractor level served as the dependent variable for statistical analyses.

### Spatial Discrimination Task

**Figure 1:**
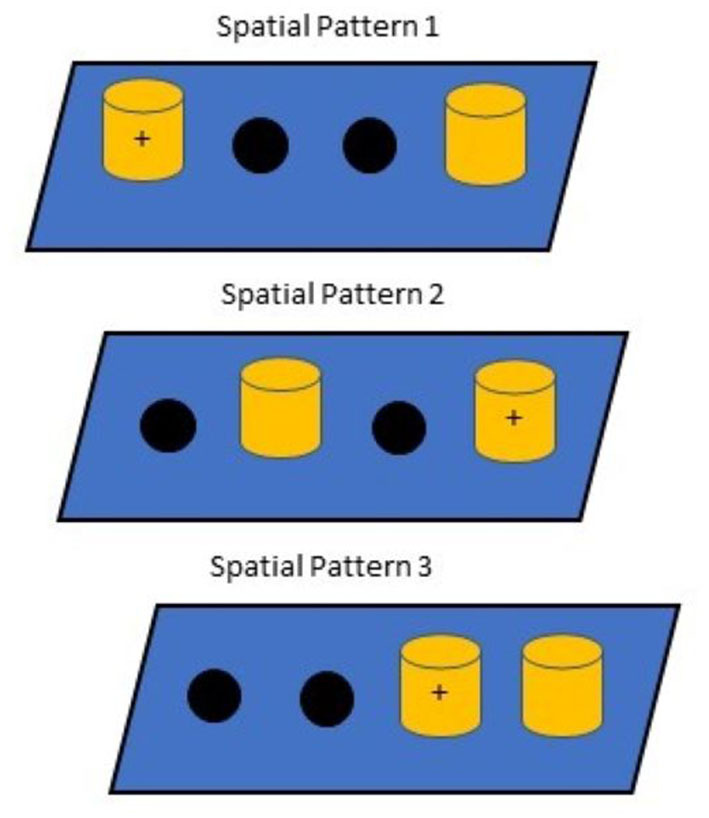
Spatial Discrimination Patterns. Correct responses are indicated as (+), which intentionally ensured that no position was correct for more than one problem. Pattern presentation occurred in the same order each day.

Spatial discrimination learning and reversal, which assesses episodic memory and executive function, respectively (46), were performed during the treatment phase only for a maximum of 41 trials, following 86 days of dosing. The task used two identical objects (i.e., red blocks) and three different spatial patterns (see Figure 1 for patterns used). During the initial training, subjects were given 15 successive trials on each pattern for a total of 45 trials. Testing continued until subjects responded correctly on 36/45 trials (80%) over a 45-trial session or responded correctly on 10 out of 15 trials (66.6%) for all three patterns within a 45-trial session. After completing the learning phase of the task, the location of the correct object for each pattern was reversed and subjects were tested until the same learning criterion was reached or until the maximum number of sessions was reached. Cumulative errors for each pattern served as the dependent variable for statistical analyses.

### Brain Imaging

Magnetic resonance spectroscopy (MRS) was performed during baseline and again at study conclusion. Single rectangular voxel (~1 cc) proton MRS was acquired at three sites: the hippocampus, frontal cortex, and cerebellum. Images were obtained using a Siemens Biograph mMR integrated 3T PET MR system. The echo time (TE) was 30 ms, repetition time (TR) 2,000 ms, averages 8, and acquisition bandwidth 2,500 Hz. Each region if interest was 8 x 8 x 15 mm and the MR technologist placing the region of interest was trained in correct placement by a veterinary radiologist. Magnetic resonance spectra were fitted to basis spectra using LCModel software. Individual peaks were included where the standard deviation of the error associated with the area under the peak was <20%. The ratio of each metabolite was calculated to total choline. A full list of the metabolites assessed can be found in Supplementary Table 1.

Imaging procedures were performed under general anesthesia. An intravenous catheter was preplaced in a cephalic vein and used for the administration of anesthetic agents and maintenance fluids. Anesthesia was induced by intravenous injection of 7 mg/kg propofol and maintained using 1.5–2% isoflurane in oxygen following endotracheal intubation. Normal saline was given at a maintenance rate of 5 ml/kg/h during anesthesia and heart rate, respiratory rate, blood pressure, and end tidal CO_2_ concentration were monitored.

### Statistical Analysis

The cognitive data were analyzed using the Statistica 11 (Tulsa, OK) statistical software with *p* values set to 0.05. Normal distribution was assessed using the Kolmogorov-Smirnov test and all tasks except for one subtask met the criterion for normality assumption. Consequently, the variables were analyzed using a repeated-measures analysis of variance (ANOVA) due to its robustness. (47) Treatment group served as a between-subject measure. For the analysis of DNMP data, treatment time-point (baseline and treatment) and delay (20s and 90s) served as within-subject variables. For the discrimination learning and reversal learning data, test (discrimination learning and reversal learning) served as a within-subject variable. For the selective attention data, treatment time-point (baseline and treatment) and distractor number (4 factors) served as within-subject measures. For the spatial discrimination task, phase (learning and reversal) and pattern (3 factors) served as within-subject variables. Retention of the discrimination learning test was analyzed using an independent samples *t*-test.

The MRS data were analyzed using MATLAB Software (The MathWorks, Inc.). At both imaging time points, the data for each treatment group were tested for normality using the Shapiro-Wilk test. Data were then tested for equality of variances using either Bartlett's test (normal data) or the Brown-Forsythe test (non-normal data). Based on the results, omnibus group comparisons for every fixed time point were conducted using either the parametric ANOVA test or the non-parametric Kruskal-Wallis test. For significant study-level assessments, *post-hoc* pairwise group comparisons were made using independent samples *t*-tests (for normally distributed data) or Wilcoxon Ranked Sum tests (for non-normally distributed data). The false discovery rate correction was applied to the resulting *p*-values. Adjusted *p*-values < 0.05 were reported as significant.

## Results

### Group Differences at Baseline

Ages of the animals ranged from 6.5 to 13.9 years in both treatment groups (μ ± SD = 8.08 ± 2.34; median = 7.15 in the supplemented group and μ ± SD = 8.85 ± 2.42; median = 8.00 in the placebo group) and no group differences were evident on any of the baseline assessments, indicating the groups were sufficiently balanced on all three cognitive measures. Mean % correct on 20s DNMP, mean % correct on 90s DNMP, mean cumulative errors on discrimination, and mean % correct on attention were 75.42, 63.33, 34.88, and 86.20 in the supplement group, respectively, and 74.58, 63.47, 34.75, and 88.49 in the placebo group, respectively. *T*-test results for each task were *p* = 0.80, 1.70, 6.07, and 1.83, respectively.

### DNMP

**Figure 2:**
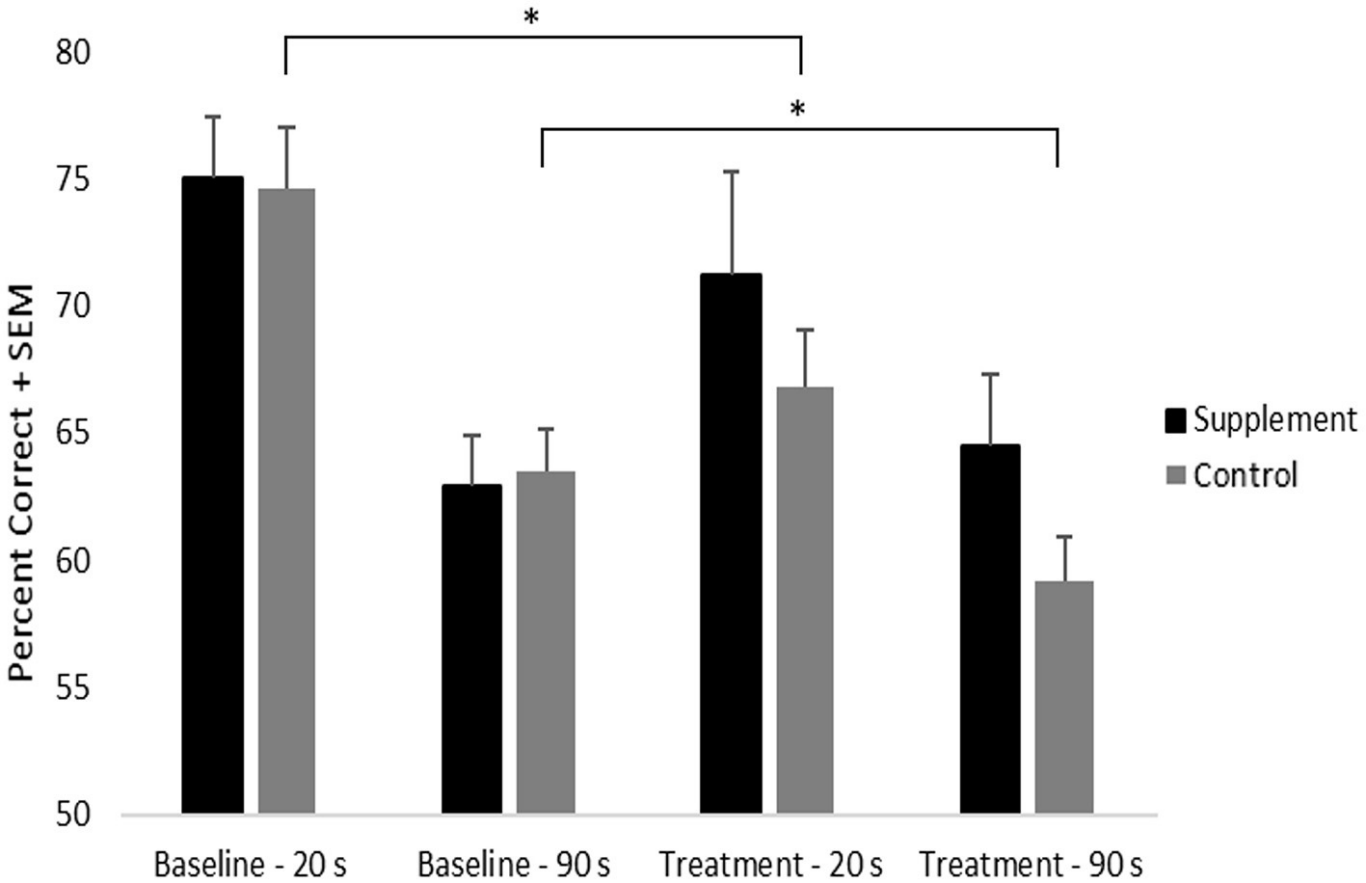
DNMP performance by treatment group, time-point, and delay. An * indicates significant difference (*p* = 0.02) from baseline to treatment in the control group, independent of delay.

Percent correct on DNMP at each delay was analyzed using a repeated-measures ANOVA with treatment group serving as a between-subject variable and with delay and treatment time-point serving as within-subject measures (Figure 2). Significant main effects of treatment time-point [*F*_(1,21)_ = 4.6; *p* = 0.043] and delay [*F*_(1,21)_ = 58.1; *p* < 0.001] were found as was a significant interaction between treatment time-point and delay [*F*_(1,21)_ = 5.9; *p* = 0.02]. The time-point effect was due to significantly lower overall performance during the treatment phase compared to baseline and the delay effect was due to lower overall performance following the long delay (90s) compared to the short delay (20s). The interaction between treatment time-point and delay indicated that progressive memory decline was most evident at the short delay, although performance at the long delay was also lower at the treatment time-point compared to baseline.

Although the treatment time-point by group interaction did not reach statistical significance, a significant decline (*p* = 0.02) in performance from baseline to treatment was found in the placebo group. By contrast, the supplemented group did not show performance decline from baseline to treatment. This suggests that the progressive decline in memory performance seen in the control group may have been attenuated by the supplement (Figure 2). No significant treatment group differences were noted at either time point or at either delay.

### Discrimination Learning, Retention and Reversal Testing

**Figure 3:**
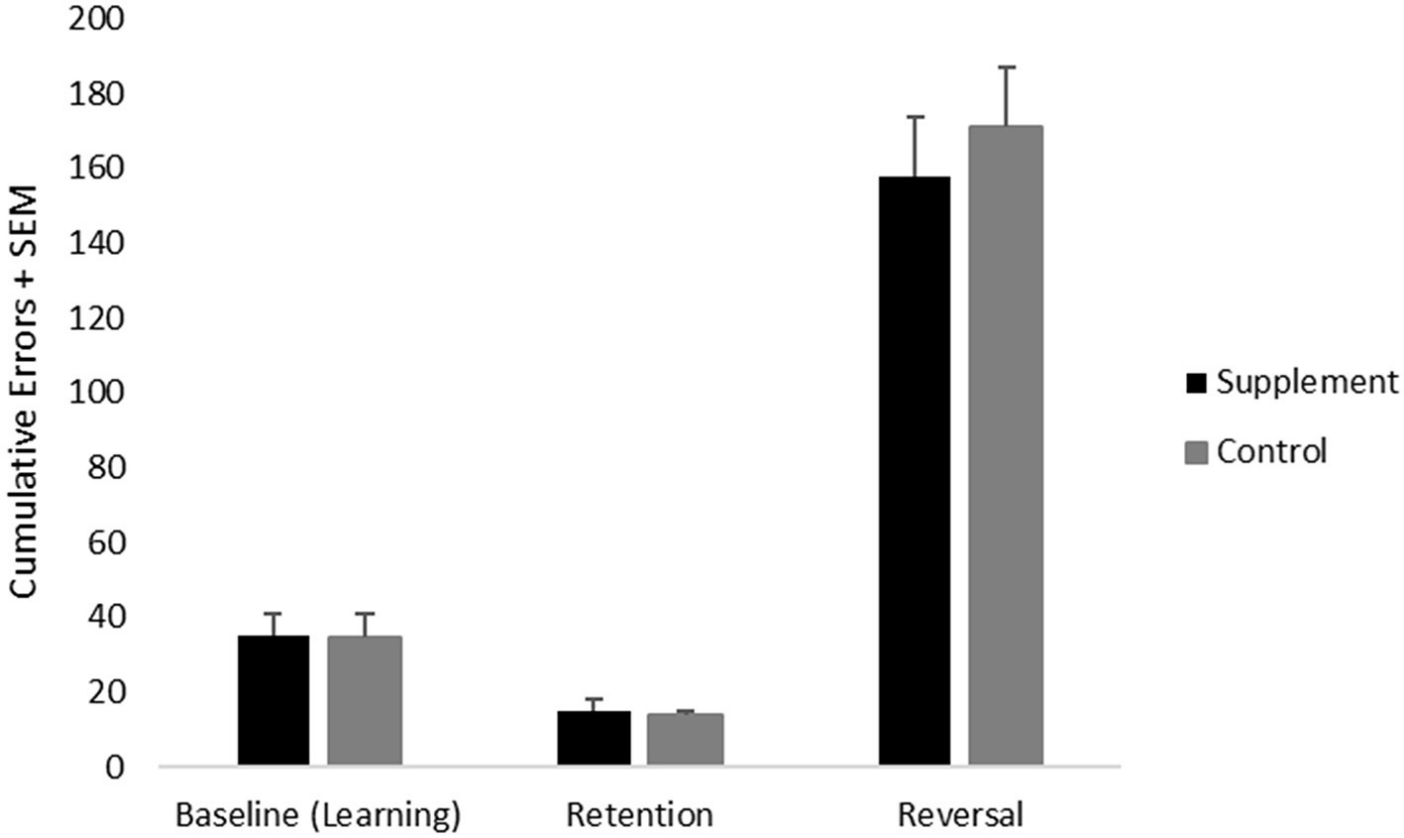
Mean cumulative errors on discrimination learning (performed at baseline) as well as retention and reversal tasks (both performed during the treatment phase) across treatment groups.

Cumulative errors on the discrimination learning and reversal task were analyzed using a repeated-measures ANOVA with treatment group as a between-subject variable and test (learning vs. reversal) as a within-subject measure. Consistent with previous findings, there was a significant [(*F*_(1,20)_ = 188.9; *p* < 0.001] main effect of test reflecting significantly higher cumulative errors on reversal learning compared to learning (Figure 3). No treatment group related effects were found.

Retention of the discrimination learning test, which provides a measure of long-term memory, was analyzed using an independent samples *t*-test. There were no significant treatment group effects.

### Selective Attention

**Figure 4:**
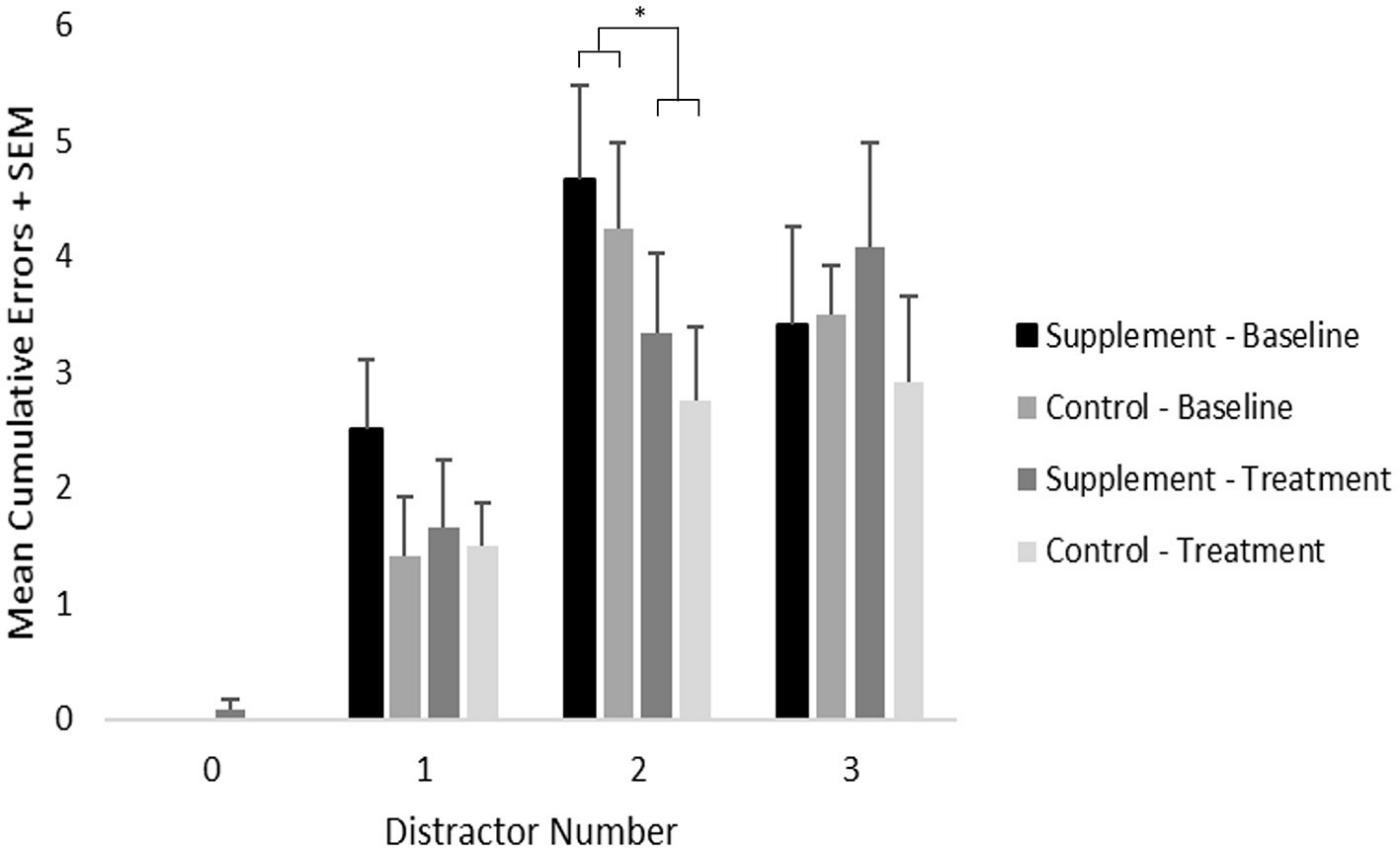
Mean cumulative errors on the selective attention task by distractor number, treatment group and time-point. As indicated by an ^*^, significant (*p* = 0.003) overall improvement was found under the 2-distractor condition; however, no treatment group effects were found.

Mean cumulative errors for each distractor number was analyzed using a repeated-measures ANOVA with treatment group as a between-subject variable and with treatment time-point (baseline vs. treatment) and distractor number serving as within-subject variables (Figure 4). The zero-distractor condition was not included in the analysis because nearly all subjects performed perfectly. A significant main effect of distractor number was found [*F*_(2,44)_ = 25.7; *p* < 0.001], which reflected significantly increased errors under the two (*p* < 0.001) and three (*p* < 0.001) distractor conditions compared to the single distractor condition when including data from both treatment groups at both testing time-points. A trend [*F*_(2,44)_ = 2.9; *p* = 0.07] for an interaction between treatment time-point and distractor number was also found, which reflected a significant overall improvement (p=0.003) from baseline to treatment on the two distracter condition only, which may have reflected a practice effect independent of treatment condition. No main effects or interactions related to treatment group were found. Although additional *post-hoc* analyses suggested improved performance on the single distractor condition in the supplemented group, this did not reach statistical significance.

### Spatial Discrimination

Cumulative errors on the learning and reversal phases of this task across each pattern were analyzed using a repeated-measures ANOVA with treatment group as a between-subject variable, and with test phase (learning and reversal) and pattern (3 factors) as within-subject variables. A significant [*F*_(2,44)_ = 34.2; *p* < 0.001] main effect of pattern and a significant [*F*_(2,44)_ = 9.4; *p* < 0.001] test phase by pattern interaction were found when including data from both treatment groups at both testing time-points. The pattern effect reflected significantly more errors (*p* < 0.001) committed on pattern 2 compared to the other patterns, whereas the test phase by pattern interaction reflected significantly more errors (*p* < 0.001) committed on pattern 3 during the reversal phase compared to the learning phase. A trend for a pattern by treatment group [*F*_(2,44)_ = 2.4; *p* = 0.10] interaction was found, which reflected significantly lower errors in the supplemented group compared to placebo during both learning (*p* = 0.01) and reversal (*p* = 0.01) of pattern 2, which was the most difficult pattern. Moreover, lower cumulative errors were seen in the supplemented group compared to the placebo group generally across patterns and task phase, although this did not reach statistical significance.

**Figure 5:**
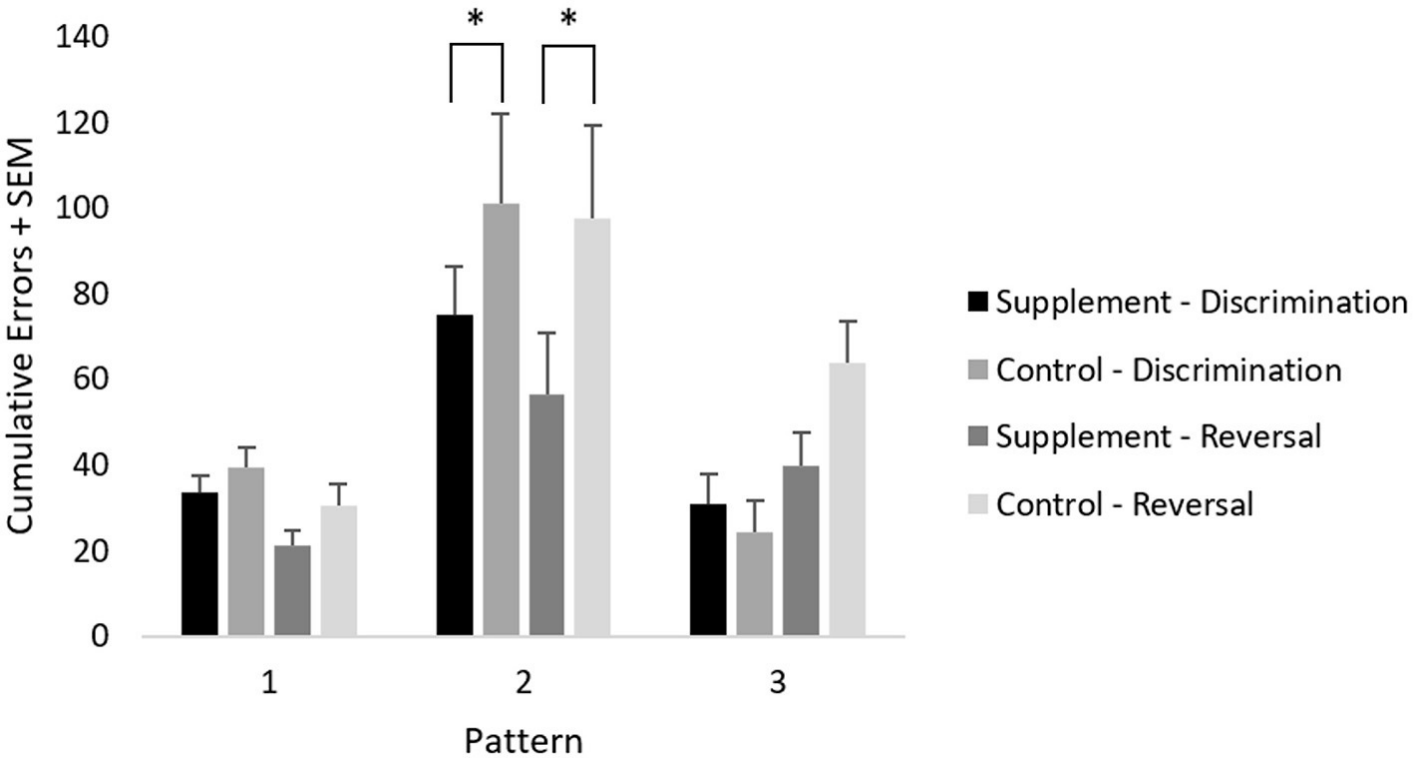
Mean cumulative errors on spatial discrimination across patterns and test phases. An * represents significantly lower errors (*p* = 0.01) in the supplement group compared to the control group on pattern 2, independently of task phase.

Although not evident in a specific interaction, the significant reversal learning deficit on pattern 3 was most likely attributed to the placebo group, which showed a substantially larger increase in errors on the reversal phase compared to the learning phase; specifically, the supplemented group showed less of an increase in errors from baseline to treatment, although this did not reach statistical significance (Figure 5).

### Brain Imaging

A list of all metabolites assessed and the average values for each group can be found in Supplementary Table 1. The MRS data revealed two significant findings. The first was that frontal lobe glutamate plus glutamine (Glu+Gln) was significantly (*p* = 0.0486) higher following treatment with the supplement compared to placebo, whereas no group differences were evident at baseline, although, neither group showed significant changes from baseline to the treatment phase. The second significant finding (*p* = 0.0285) was that hippocampal glycerophosphocholine plus phosphocholine (GPC+PCh) was increased in the supplemented group compared to baseline; although, there were no treatment group differences either at baseline or following treatment.

The data acquired in this study demonstrated that some of the metabolites did not meet the conventional criterion for acceptance as valid data; specifically, the area under the peak did not meet 20% error estimate for individual metabolites in each subject. To obtain quality data, the magnetic field within the voxel must be as homogeneous as possible. Fewer of the data points met this criterion in the frontal lobe compared to the hippocampus and cerebellum. The cone shape of the skull in this region and the adjacent large frontal sinuses made shimming much more challenging and this most likely accounted for the poorer data quality associated with the frontal lobe voxel.

## Discussion

The current study examined the effects of a novel lipid supplement combining sphingolipids and DHA on several measures of cognitive function as well as on brain metabolism assessed using MRS imaging compared to placebo control in aged Beagle dogs. Specifically, the experiment employed a battery of cognitive tasks that assessed diverse cognitive domains (1, 2, 4, 13, 14) impaired in canine aging. Results from a number of the tasks (i.e., DNMP, spatial discrimination learning, and spatial discrimination reversal) suggest treatment with the supplement may provide cognitive benefits, including attenuation of working memory decline, improved episodic memory, and improved executive function, although, treatment effects were not evident on memory retention or one of the two executive function related tasks (i.e., discrimination retention and discrimination reversal).

Performance on the DNMP task, which is impaired early in canine aging and is used to evaluate working memory, has previously been shown to decline over time (1, 4). In this experiment, the control group showed a decline in DNMP performance from baseline to the treatment time-point, which is consistent with what has been previously reported and may reflect a cognitive measure of the neurodegenerative processes occurring in Beagle dogs; however, the supplemented group did not experience this time-dependent decline in DNMP performance in the current study. Previous lesion studies examining the regional basis of DNMP performance in dogs demonstrate that the DNMP task, but not long-term memory, is disrupted by dorsolateral prefrontal cortical lesions (48). Moreover, impaired performance on the DNMP is inversely correlated with prefrontal amyloid load and frontal lobe atrophy (49). Thus, one hypothesis is that the preservation of DNMP performance seen following supplementation could reflect an attenuation of frontocortical neuropathological processes.

Similarly, loss of selective processes of pattern separation associated with episodic memory (46) as well as reversal learning deficits associated with executive dysfunction are consistently found in aged dogs (2, 14), the latter of which are also linked to amyloidosis and atrophy of the frontal cortex. (49) In this study, the supplemented group committed a reduced number of errors on pattern 2 of the spatial discrimination task, which was determined to be the most difficult pattern in this study as well as in previous research (46). Moreover, the supplemented group generally committed fewer errors across all patterns of the task compared to placebo, although this was not statistically significant. These findings could indicate attenuation of decline in episodic memory related to hippocampal processes. Consistent with improvement in executive function, the supplemented group also showed improved reversal learning on one pattern of the spatial discrimination task. The specific tasks in which the supplemented group statistically outperformed the control group suggest that the novel lipid extract may attenuate general short-term working memory deficits and increase episodic memory and executive function in impaired subjects. On the other hand, a significant treatment effect on discrimination reversal learning was not found, which suggests the finding on the spatial discrimination reversal may be a spurious result or may reflect cognitive-domain specific differences underlying the two reversal tests.

MRS imaging can be used in dogs to detect both regional and age-related differences of metabolites, although more research is required to standardize imaging protocols (50–52). In the current study, MRS quantification of brain metabolites indicated that frontal lobe Glu and Gln were greater in the supplemented group compared to control; however, neither group showed significant changes from baseline to the treatment phase. Previous literature suggests a decline in Glu and Gln is correlated with cognitive decline in Alzheimer's disease as well as in aged dogs compared to younger cohorts (53–57). This finding may support the hypothesis that frontal cortical function was improved by the test product, specifically frontal lobe pathology including amyloidosis and frontal cortical atrophy, which occurs early in canine aging and is linked to executive function and working memory deficits (20, 58). This is consistent with the performance improvements on the DNMP and spatial discrimination tasks seen in supplemented group; however, because no significant difference was found when comparing baseline to the end of the treatment period, it is possible the finding is related to individual animal differences or is a spurious effect. Given that a number of the imaging data points from the frontal lobe did not meet the conventional criterion for acceptance as valid data, additional studies are needed to confirm the relationship of the supplement to Glu+Gln as well as to improve MRS procedures in dogs as many technical factors must be considered to optimize imaging quality (50, 51).

A significant increase in hippocampal GPC+PCh was also found from baseline to treatment in the supplemented group; however, no differences to the control group were found either at baseline or following treatment. Although increased GPC+PCh is associated with greater breakdown of cell membranes as well as cognitive decline and neurodegeneration (59), the increase may also reflect sphingolipid-induced changes in lipid metabolism. Further work is required to determine the cause of the increases in GPC+PCh and whether this finding reflects a negative or potentially positive effect of the supplement.

Previous studies evaluating the effects of DHA on cognitive development in puppies indicate that DHA dose-dependently improves performance across multiple cognitive domains (37). By contrast, the effects of DHA supplementation on cognitive performance in aged dogs have been less impressive; improved contrast sensitivity has been attributed to DHA supplementation but working memory performance and complex learning were not affected in the same study (36). In the current study, the combined DHA and sphingolipid supplementation may have attenuated longitudinal DNMP performance decline and potentially improved some measures of executive dysfunction that are hallmarks of age-related canine cognitive deficits preceding behavioral signs associated with CDS (14). A study evaluating a combination of DHA and pig-derived phospholipids, which included sphingomyelin, suggested the supplement improved working memory performance and quality of life in aged dogs (19). We cannot directly compare results among the above studies because the protocols differed; however, the results of the current study suggest that the supplement improved cognitive performance on a subset of the cognitive domains evaluated here, which suggests that the addition of porcine brain-derived sphingolipids may provide additive or synergistic benefit to the effects of DHA previously seen (36). Confirmatory studies with greater sample sizes are warranted to better establish the benefits of the current supplement on cognitive measures and additional doses of the supplement should be examined to better confirm specificity of effects.

In conclusion, the group receiving the test supplement demonstrated improved performance across a subset of cognitive tasks on which performance is impaired in canine aging. The attenuation of progressive memory decline assessed with the DNMP in combination with the findings of increased Glu and Gln in the frontal cortex may suggest the supplement ameliorates frontal lobe deficits impaired early in the course of canine cognitive aging. Improvements on both reversal tasks would further support this hypothesis, however, only performance on a subtest on the spatial discrimination reversal was improved by treatment. Based on the current results, further development of this novel lipid supplement for attenuating cognitive decline or improving cognitive function during development is warranted in both dogs and humans. Limitations of the current study, which could be improved upon in future research, include use of a larger sample size to increase power, equal representation of male and female subjects, histopathological analysis of brain samples, implementation of a crossover design to determine the duration of effects following discontinuation of supplement administration, and a longer treatment period, which may result in an even greater decline in DNMP performance in untreated animals.

## Data Availability Statement

The raw data supporting the conclusions of this article will be made available by the authors, without undue reservation.

## Ethics Statement

The animal study was reviewed and approved by InterVivo Solutions Inc. Animal Care and Use Committee. Animal maintenance and experimental procedures were performed in accordance with the guidelines of the Ontario Ministry of Agriculture Food and Rural Affairs (OMAFRA), the Canadian Council on Animal Care (CCAC) and the Institutional Animal Care and Use Committee (IACUC).

## Author Contributions

JA, NM, and SS contributed to the conception and design of the study as well as interpretation of the data. JM composed the first draft of the manuscript. AP and MB were responsible for data acquisition. ST performed statistical analysis. All authors contributed to manuscript revision, read, and approved submitted version.

## Funding

This study was funded through contract from Bioiberica S.A.U. to InterVivo Solutions Inc.

## Conflict of Interest

SS is employed by Bioiberica S.A.U., Esplugues de Llobregat, Spain. JA, JM, AP, MB, and ST were employed by the contract research organization that conducted the study (InterVivo Solutions Inc.). NM is employed by CanCog Inc. The authors declare that this study received funding from Bioiberica S.A.U. The funder was involved in manufacturing of the test product, study design, preparation of the manuscript, and decision to submit the manuscript for publication.

## Publisher's Note

All claims expressed in this article are solely those of the authors and do not necessarily represent those of their affiliated organizations, or those of the publisher, the editors and the reviewers. Any product that may be evaluated in this article, or claim that may be made by its manufacturer, is not guaranteed or endorsed by the publisher.
