# Supplementary material for: Sphingolipids and DHA Improve Cognitive Deficits in Aged Beagle Dogs
Source: Front Vet Sci. 2022 Jul 13;9:646451. doi: 10.3389/fvets.2022.646451 (PMC9329143; doi:10.3389/fvets.2022.646451)
Supplement: Supplementary file 1 [file Table_1.docx]

Supplementary Material

Supplementary Table 1: Group Averages for MRS Imaging Metabolites

| **Abbreviation** | **Metabolite Name** | **Abbreviation** | **Metabolite Name** |
| --- | --- | --- | --- |
| Ala | L-Alanine | Lip09 | Lipids – 0.9 ppm |
| Asp | Aspartate | Lip13 | Lipids – 1.3 ppm |
| Cr | Creatine | Lip20 | Lipids – 2.0 ppm |
| CrCH2 | Creatine methylene | MM09 | Macromolecules – 0.9 ppm |
| GABA | Gamma-aminobutyric acid | MM12 | Macromolecules – 1.2 ppm |
| Glc | Glucose | MM14 | Macromolecules – 1.4 ppm |
| Gln | Glutamine | MM17 | Macromolecules – 1.7 ppm |
| Glu | Glutamate | MM20 | Macromolecules – 2.0 ppm |
| GPC | Glycerophosphocholine | NAA | N-Acetylaspartate |
| GSH | Glutathione | NAAG | N-Acetylaspartylglutamate |
| Ins, mI | myo-Inositol | PCh | Phosphocholine |
| Lac | L-Lactate | PCr | Phosphocreatine |
| Lac | Lactate | Scyllo | scyllo-Inositol |
|  |  | Tau | Taurine |

| **Parameter** | **Group Averages** | | | |
| --- | --- | --- | --- | --- |
|  | **Supplement Group** | | **Control Group** | |
|  | **Baseline** | **Treatment** | **Baseline** | **Treatment** |
| **Cerebellum** | | | | |
| Ala Concentration | 9.23E-06 | 1.12E-05 | 1.03E-05 | 5.76E-06 |
| Ala Estimation Error (%SD) | 570.9167 | 323.0909 | 337.3333 | 656.6667 |
| Ala Concentration Ratio to Total Ch | 0.5245 | 0.843091 | 0.669167 | 0.341417 |
| Asp Concentration | 4.06E-06 | 4.57E-06 | 4.5E-06 | 3.06E-06 |
| Asp Estimation Error (%SD) | 545.5833 | 666.1818 | 462.6667 | 666.25 |
| Asp Concentration Ratio to Total Ch | 0.737583 | 0.337364 | 0.285583 | 0.166917 |
| Cr Concentration | 2.48E-05 | 2.97E-05 | 2.74E-05 | 4.03E-05 |
| Cr Estimation Error (%SD) | 310.9167 | 298.6364 | 356.1667 | 66.33333 |
| Cr Concentration Ratio to Total Ch | 1.636167 | 2.463182 | 1.758667 | 2.377333 |
| Cr+PCr Concentration | 4.8E-05 | 4.8E-05 | 5.2E-05 | 5.62E-05 |
| Cr+PCr Estimation Error (%SD) | 92.16667 | 101 | 9.666667 | 7.5 |
| Cr+PCr Concentration Ratio to Total Ch | 2.8725 | 3.836273 | 3.1565 | 3.3515 |
| CrCH2 Concentration | 1.31E-05 | 9.46E-07 | 4.24E-06 | 1.14E-05 |
| CrCH2 Estimation Error (%SD) | 385.6667 | 750.3636 | 568 | 387.5833 |
| CrCH2 Concentration Ratio to Total Ch | 0.779417 | 0.078818 | 0.261083 | 0.635 |
| GABA Concentration | 4.98E-06 | 3.95E-06 | 5.74E-06 | 6.03E-06 |
| GABA Estimation Error (%SD) | 198.75 | 413.4545 | 99.5 | 204.75 |
| GABA Concentration Ratio to Total Ch | 0.307417 | 0.295182 | 0.3515 | 0.352167 |
| GPC Concentration | 1.18E-05 | 1.23E-05 | 1.21E-05 | 1.42E-05 |
| GPC Estimation Error (%SD) | 209.3333 | 112.4545 | 256.9167 | 102.4167 |
| GPC Concentration Ratio to Total Ch | 0.68375 | 0.896545 | 0.75 | 0.829667 |
| GPC+PCh Concentration | 1.55E-05 | 1.25E-05 | 1.48E-05 | 1.7E-05 |
| GPC+PCh Estimation Error (%SD) | 17.08333 | 110 | 93.25 | 8.666667 |
| GPC+PCh Concentration Ratio to Total Ch | 1 | 0.909091 | 0.916667 | 1 |
| GSH Concentration | 8.93E-06 | 3.37E-05 | 7.97E-06 | 7.64E-06 |
| GSH Estimation Error (%SD) | 219.1667 | 48.72727 | 305.5833 | 189.5833 |
| GSH Concentration Ratio to Total Ch | 1.19875 | 1.039273 | 0.47275 | 0.455083 |
| Glc Concentration | 6.38E-06 | 1.25E-05 | 7.44E-06 | 1.14E-05 |
| Glc Estimation Error (%SD) | 189.5 | 131.1818 | 140.0833 | 83.41667 |
| Glc Concentration Ratio to Total Ch | 1.186917 | 0.928091 | 0.452333 | 0.696833 |
| Gln Concentration | 3.89E-05 | 4.79E-05 | 2.6E-05 | 3.77E-05 |
| Gln Estimation Error (%SD) | 53.16667 | 46.63636 | 201.8333 | 36.58333 |
| Gln Concentration Ratio to Total Ch | 2.659083 | 2.714 | 1.4595 | 2.241583 |
| Glu Concentration | 3.83E-05 | 6.95E-05 | 5.53E-05 | 5.78E-05 |
| Glu Estimation Error (%SD) | 155.5833 | 31.63636 | 104.1667 | 21.41667 |
| Glu Concentration Ratio to Total Ch | 2.509 | 4.331273 | 3.128583 | 3.45925 |
| Glu+Gln Concentration | 7.72E-05 | 0.000117 | 8.13E-05 | 9.56E-05 |
| Glu+Gln Estimation Error (%SD) | 37.66667 | 18.27273 | 99.75 | 13.16667 |
| Glu+Gln Concentration Ratio to Total Ch | 5.167917 | 7.045182 | 4.588083 | 5.700667 |
| Ins Concentration | 6.31E-05 | 0.000119 | 6.04E-05 | 6.95E-05 |
| Ins Estimation Error (%SD) | 91.58333 | 9.545455 | 99.83333 | 7.5 |
| Ins Concentration Ratio to Total Ch | 3.9205 | 5.174545 | 3.8235 | 4.12775 |
| Lac Concentration | 7.56E-06 | 5.72E-06 | 7.21E-06 | 6.33E-06 |
| Lac Estimation Error (%SD) | 523.5833 | 612.2727 | 502.1667 | 532.9167 |
| Lac Concentration Ratio to Total Ch | 0.454667 | 0.569 | 0.44625 | 0.372167 |
| Lip09 Concentration | 1.07E-05 | 6.7E-06 | 5.81E-06 | 6.59E-06 |
| Lip09 Estimation Error (%SD) | 617.9167 | 559.7273 | 805.1667 | 488.9167 |
| Lip09 Concentration Ratio to Total Ch | 22.06867 | 0.539727 | 0.081983 | 0.397335 |
| Lip13a Concentration | 4.17E-05 | 1.95E-05 | 2.04E-05 | 2.52E-05 |
| Lip13a Estimation Error (%SD) | 420.6667 | 801.0909 | 791.5833 | 489.5833 |
| Lip13a Concentration Ratio to Total Ch | 76.73392 | 1.331455 | 0.3126 | 1.524125 |
| Lip13a+Lip13b Concentration | 4.54E-05 | 2.87E-05 | 2.46E-05 | 2.91E-05 |
| Lip13a+Lip13b Estimation Error (%SD) | 258.5833 | 368.2727 | 571.4167 | 310.75 |
| Lip13a+Lip13b Concentration Ratio to Total Ch | 76.90667 | 2.071165 | 0.5636 | 1.752292 |
| Lip13b Concentration | 3.63E-06 | 9.23E-06 | 4.14E-06 | 3.97E-06 |
| Lip13b Estimation Error (%SD) | 836.9167 | 524 | 778.8333 | 731 |
| Lip13b Concentration Ratio to Total Ch | 0.17275 | 0.73971 | 0.251 | 0.228167 |
| Lip20 Concentration | 6.84E-06 | 3.09E-06 | 1.36E-06 | 3.85E-06 |
| Lip20 Estimation Error (%SD) | 442.75 | 508.2727 | 748.8333 | 353.4167 |
| Lip20 Concentration Ratio to Total Ch | 13.13038 | 0.234094 | 0.081833 | 0.23125 |
| MM09 Concentration | 6.24E-05 | 3.41E-05 | 4.29E-05 | 5.1E-05 |
| MM09 Estimation Error (%SD) | 19.83333 | 121.8182 | 105.6667 | 18.25 |
| MM09 Concentration Ratio to Total Ch | 18.41383 | 2.995 | 2.703083 | 3.00325 |
| MM09+Lip09 Concentration | 7.31E-05 | 4.08E-05 | 4.87E-05 | 5.76E-05 |
| MM09+Lip09 Estimation Error (%SD) | 15.83333 | 109.2727 | 21 | 13.75 |
| MM09+Lip09 Concentration Ratio to Total Ch | 40.4825 | 3.534545 | 2.784917 | 3.40075 |
| MM12 Concentration | 2.14E-05 | 8.99E-06 | 1.3E-05 | 1.48E-05 |
| MM12 Estimation Error (%SD) | 118 | 157.8182 | 89.25 | 69.75 |
| MM12 Concentration Ratio to Total Ch | 8.39275 | 0.825182 | 0.81375 | 0.86875 |
| MM14 Concentration | 5.14E-05 | 2.05E-05 | 3.97E-05 | 4.76E-05 |
| MM14 Estimation Error (%SD) | 121.25 | 279.2727 | 131.3333 | 44.41667 |
| MM14 Concentration Ratio to Total Ch | 3.259 | 1.861545 | 2.499917 | 2.816167 |
| MM14+Lip13a+L Concentration | 0.000118 | 5.83E-05 | 7.73E-05 | 9.16E-05 |
| MM14+Lip13a+L Estimation Error (%SD) | 28.41667 | 64.54545 | 38.5 | 27.58333 |
| MM14+Lip13a+L Concentration Ratio to Total Ch | 88.55842 | 4.758182 | 3.87725 | 5.437 |
| MM17 Concentration | 5.23E-05 | 1.64E-05 | 3.24E-05 | 4.33E-05 |
| MM17 Estimation Error (%SD) | 185.0833 | 476.5455 | 53.16667 | 23.58333 |
| MM17 Concentration Ratio to Total Ch | 32.08633 | 1.301636 | 1.817 | 2.562917 |
| MM20 Concentration | 8.71E-05 | 5.04E-05 | 6.2E-05 | 7.37E-05 |
| MM20 Estimation Error (%SD) | 127.8333 | 214 | 117.6667 | 36.75 |
| MM20 Concentration Ratio to Total Ch | 5.681667 | 4.439 | 3.936 | 4.349917 |
| MM20+Lip20 Concentration | 9.4E-05 | 5.35E-05 | 6.33E-05 | 7.76E-05 |
| MM20+Lip20 Estimation Error (%SD) | 43.83333 | 79.36364 | 115.9167 | 33.41667 |
| MM20+Lip20 Concentration Ratio to Total Ch | 18.81208 | 4.673091 | 4.01775 | 4.581083 |
| NAA Concentration | 2.16E-05 | 2.7E-05 | 3.45E-05 | 3.34E-05 |
| NAA Estimation Error (%SD) | 127.5833 | 131.5455 | 120.5 | 110.1667 |
| NAA Concentration Ratio to Total Ch | 1.2805 | 2.022182 | 2.124417 | 1.924 |
| NAA+NAAG Concentration | 4.66E-05 | 4.93E-05 | 5.65E-05 | 5.91E-05 |
| NAA+NAAG Estimation Error (%SD) | 18.25 | 103.9091 | 93.66667 | 10.83333 |
| NAA+NAAG Concentration Ratio to Total Ch | 6.300167 | 4.034182 | 3.531917 | 3.512333 |
| NAAG Concentration | 2.49E-05 | 2.23E-05 | 2.21E-05 | 2.57E-05 |
| NAAG Estimation Error (%SD) | 47 | 132.4545 | 126.6667 | 121.4167 |
| NAAG Concentration Ratio to Total Ch | 5.01975 | 2.011727 | 1.4075 | 1.588333 |
| PCh Concentration | 3.7E-06 | 2.06E-07 | 2.73E-06 | 2.83E-06 |
| PCh Estimation Error (%SD) | 494 | 930.1818 | 835.3333 | 738.4167 |
| PCh Concentration Ratio to Total Ch | 0.31625 | 0.012545 | 0.166667 | 0.170333 |
| PCr Concentration | 2.32E-05 | 1.83E-05 | 2.46E-05 | 1.59E-05 |
| PCr Estimation Error (%SD) | 446.5 | 413.1818 | 215.3333 | 456.3333 |
| PCr Concentration Ratio to Total Ch | 1.23625 | 1.373091 | 1.398 | 0.974 |
| Scyllo Concentration | 1.02E-06 | 4.92E-07 | 8.32E-07 | 1.1E-06 |
| Scyllo Estimation Error (%SD) | 306.75 | 551.9091 | 413 | 279.6667 |
| Scyllo Concentration Ratio to Total Ch | 0.326333 | 0.047191 | 0.05225 | 0.064 |
| Tau Concentration | 5.28E-06 | 3.43E-06 | 4.66E-06 | 4.64E-06 |
| Tau Estimation Error (%SD) | 338.75 | 591 | 415.5833 | 488.1667 |
| Tau Concentration Ratio to Total Ch | 1.767 | 0.339182 | 0.28625 | 0.260417 |
| **Frontal** | | | | |
| Ala Concentration | 1.32E-05 | 1.23E-05 | 1.09E-05 | 2.96E-05 |
| Ala Estimation Error (%SD) | 95.83333 | 328.1818 | 267 | 219.9167 |
| Ala Concentration Ratio to Total Ch | 1.203083 | 0.606091 | 0.573 | 0.818667 |
| Asp Concentration | 6.42E-06 | 7.93E-06 | 2.79E-06 | 3.65E-06 |
| Asp Estimation Error (%SD) | 451.4167 | 420.4545 | 641.25 | 523.1667 |
| Asp Concentration Ratio to Total Ch | 0.319333 | 0.341909 | 0.149117 | 0.196333 |
| Cr Concentration | 2.88E-05 | 7.6E-05 | 3.77E-05 | 4.06E-05 |
| Cr Estimation Error (%SD) | 271.3333 | 289.3636 | 266.9167 | 295.8333 |
| Cr Concentration Ratio to Total Ch | 1.676333 | 2.615636 | 1.899917 | 1.798667 |
| Cr+PCr Concentration | 4.98E-05 | 9.29E-05 | 5.33E-05 | 5.91E-05 |
| Cr+PCr Estimation Error (%SD) | 9.75 | 106.4545 | 7.666667 | 9.583333 |
| Cr+PCr Concentration Ratio to Total Ch | 3.169417 | 3.213 | 2.9055 | 3.64475 |
| CrCH2 Concentration | 2.04E-05 | 7.19E-06 | 5.31E-06 | 3.31E-06 |
| CrCH2 Estimation Error (%SD) | 519.8333 | 702 | 720.1667 | 718.9167 |
| CrCH2 Concentration Ratio to Total Ch | 0.9455 | 0.205091 | 0.300083 | 0.097917 |
| GABA Concentration | 4.52E-06 | 5.23E-06 | 7.4E-06 | 6.29E-06 |
| GABA Estimation Error (%SD) | 188.25 | 181.5455 | 109.3333 | 97.58333 |
| GABA Concentration Ratio to Total Ch | 0.3025 | 0.222 | 0.4115 | 0.353917 |
| GPC Concentration | 1.36E-05 | 1.69E-05 | 1.76E-05 | 1.52E-05 |
| GPC Estimation Error (%SD) | 179.1667 | 191.8182 | 20.5 | 184.1667 |
| GPC Concentration Ratio to Total Ch | 0.80375 | 0.818182 | 0.94 | 0.791917 |
| GPC+PCh Concentration | 1.61E-05 | 1.69E-05 | 1.84E-05 | 1.58E-05 |
| GPC+PCh Estimation Error (%SD) | 10.75 | 191.8182 | 7.75 | 97.08333 |
| GPC+PCh Concentration Ratio to Total Ch | 1 | 0.818182 | 1 | 0.916667 |
| GSH Concentration | 1.41E-05 | 4.68E-05 | 8.78E-06 | 2.9E-05 |
| GSH Estimation Error (%SD) | 281.1667 | 280.3636 | 200.1667 | 227.4167 |
| GSH Concentration Ratio to Total Ch | 0.613 | 1.735636 | 0.467 | 0.382583 |
| Glc Concentration | 3.44E-06 | 7.66E-06 | 8.76E-06 | 5.31E-06 |
| Glc Estimation Error (%SD) | 252.1667 | 82 | 290.5 | 280.8333 |
| Glc Concentration Ratio to Total Ch | 0.243 | 0.345636 | 0.438917 | 0.29875 |
| Gln Concentration | 4.42E-05 | 4.58E-05 | 4.09E-05 | 5.28E-05 |
| Gln Estimation Error (%SD) | 49.41667 | 131.7273 | 32.33333 | 118.75 |
| Gln Concentration Ratio to Total Ch | 3.103917 | 2.569455 | 2.330083 | 2.204352 |
| Glu Concentration | 4.74E-05 | 0.000114 | 7.19E-05 | 9.99E-05 |
| Glu Estimation Error (%SD) | 105.75 | 107.3636 | 15.75 | 103 |
| Glu Concentration Ratio to Total Ch | 3.646333 | 4.040909 | 3.957583 | 3.539333 |
| Glu+Gln Concentration | 9.14E-05 | 0.00016 | 0.000113 | 0.000153 |
| Glu+Gln Estimation Error (%SD) | 17.83333 | 17.09091 | 11.33333 | 96.66667 |
| Glu+Gln Concentration Ratio to Total Ch | 6.750583 | 6.610364 | 6.287583 | 5.743518 |
| Ins Concentration | 5.01E-05 | 5.54E-05 | 6.54E-05 | 8.45E-05 |
| Ins Estimation Error (%SD) | 92.08333 | 191.2727 | 8.166667 | 92.5 |
| Ins Concentration Ratio to Total Ch | 3.69 | 2.349182 | 3.558167 | 2.991167 |
| Lac Concentration | 8.12E-06 | 1.06E-05 | 5.78E-06 | 5.67E-06 |
| Lac Estimation Error (%SD) | 478.75 | 575.2727 | 574.4167 | 674.75 |
| Lac Concentration Ratio to Total Ch | 0.826083 | 0.267364 | 0.348667 | 0.342417 |
| Lip09 Concentration | 5.54E-10 | 3.13E-05 | 3.03E-06 | 5.38E-07 |
| Lip09 Estimation Error (%SD) | 999 | 656.0909 | 772.9167 | 946.5 |
| Lip09 Concentration Ratio to Total Ch | 1.61E-05 | 0.074427 | 0.150083 | 0.003 |
| Lip13a Concentration | 4.52E-06 | 1.62E-05 | 1.99E-05 | 7.41E-06 |
| Lip13a Estimation Error (%SD) | 678.5 | 621.2727 | 565.4167 | 766.25 |
| Lip13a Concentration Ratio to Total Ch | 0.385375 | 0.321545 | 1.069833 | 0.39275 |
| Lip13a+Lip13b Concentration | 4.93E-06 | 1.94E-05 | 2.13E-05 | 1.3E-05 |
| Lip13a+Lip13b Estimation Error (%SD) | 601.9167 | 429.0909 | 424.9167 | 485.0833 |
| Lip13a+Lip13b Concentration Ratio to Total Ch | 0.409625 | 0.500818 | 1.141 | 0.4895 |
| Lip13b Concentration | 4.15E-07 | 3.19E-06 | 1.35E-06 | 5.62E-06 |
| Lip13b Estimation Error (%SD) | 922.4167 | 731.0909 | 851.1667 | 717.8333 |
| Lip13b Concentration Ratio to Total Ch | 0.02425 | 0.179273 | 0.071167 | 0.09675 |
| Lip20 Concentration | 3.81E-07 | 1.89E-05 | 1.86E-06 | 1.72E-06 |
| Lip20 Estimation Error (%SD) | 869.9167 | 482.7273 | 783.8333 | 705.25 |
| Lip20 Concentration Ratio to Total Ch | 0.040453 | 0.232322 | 0.112542 | 0.056651 |
| MM09 Concentration | 2.47E-05 | 6.4E-05 | 2.92E-05 | 3.92E-05 |
| MM09 Estimation Error (%SD) | 341.4167 | 199.0909 | 88.5 | 106 |
| MM09 Concentration Ratio to Total Ch | 1.521917 | 1.634382 | 1.5465 | 4.716167 |
| MM09+Lip09 Concentration | 2.47E-05 | 9.53E-05 | 3.22E-05 | 3.98E-05 |
| MM09+Lip09 Estimation Error (%SD) | 341.4167 | 110.1818 | 36.91667 | 52.83333 |
| MM09+Lip09 Concentration Ratio to Total Ch | 1.521917 | 1.708836 | 1.696583 | 4.719083 |
| MM12 Concentration | 7.6E-06 | 1.65E-05 | 9.69E-06 | 1.2E-05 |
| MM12 Estimation Error (%SD) | 432.8333 | 311.7273 | 116.0833 | 124.5833 |
| MM12 Concentration Ratio to Total Ch | 0.473501 | 0.423752 | 0.507833 | 1.413583 |
| MM14 Concentration | 3.11E-05 | 4.82E-05 | 2.84E-05 | 3.15E-05 |
| MM14 Estimation Error (%SD) | 442.9167 | 307 | 218.6667 | 282.4167 |
| MM14 Concentration Ratio to Total Ch | 1.87325 | 1.575545 | 1.539917 | 3.933417 |
| MM14+Lip13a+L Concentration | 4.35E-05 | 8.41E-05 | 5.93E-05 | 5.65E-05 |
| MM14+Lip13a+L Estimation Error (%SD) | 268.4167 | 208.9091 | 44.33333 | 45.08333 |
| MM14+Lip13a+L Concentration Ratio to Total Ch | 2.756167 | 2.500025 | 3.18875 | 5.8365 |
| MM17 Concentration | 3.37E-05 | 0.000114 | 3.83E-05 | 4.29E-05 |
| MM17 Estimation Error (%SD) | 341.0833 | 198.5455 | 59.5 | 106.6667 |
| MM17 Concentration Ratio to Total Ch | 2.012699 | 3.233088 | 2.0305 | 3.2335 |
| MM20 Concentration | 4.32E-05 | 9.05E-05 | 4.52E-05 | 7E-05 |
| MM20 Estimation Error (%SD) | 393.8333 | 295.6364 | 95.66667 | 111.5 |
| MM20 Concentration Ratio to Total Ch | 2.651206 | 2.478391 | 2.450583 | 8.208083 |
| MM20+Lip20 Concentration | 4.36E-05 | 0.000109 | 4.71E-05 | 7.17E-05 |
| MM20+Lip20 Estimation Error (%SD) | 330.0833 | 120.4545 | 52.58333 | 42.41667 |
| MM20+Lip20 Concentration Ratio to Total Ch | 2.691545 | 2.710673 | 2.563083 | 8.264833 |
| NAA Concentration | 1.15E-05 | 1.8E-05 | 2.89E-05 | 1.58E-05 |
| NAA Estimation Error (%SD) | 369.75 | 300.4545 | 46.5 | 435.75 |
| NAA Concentration Ratio to Total Ch | 0.94425 | 0.981545 | 1.496417 | 0.85775 |
| NAA+NAAG Concentration | 5E-05 | 4.57E-05 | 5.64E-05 | 4.25E-05 |
| NAA+NAAG Estimation Error (%SD) | 13 | 109.1818 | 10.25 | 100 |
| NAA+NAAG Concentration Ratio to Total Ch | 3.346667 | 2.589182 | 3.02175 | 2.311583 |
| NAAG Concentration | 3.86E-05 | 2.76E-05 | 2.76E-05 | 2.67E-05 |
| NAAG Estimation Error (%SD) | 23.25 | 143.2727 | 111.1667 | 204.75 |
| NAAG Concentration Ratio to Total Ch | 2.402333 | 1.607545 | 1.525583 | 1.453833 |
| PCh Concentration | 2.44E-06 | 0 | 7.9E-07 | 5.4E-07 |
| PCh Estimation Error (%SD) | 763.6667 | 999 | 853.3333 | 849 |
| PCh Concentration Ratio to Total Ch | 0.19625 | 0 | 0.06 | 0.12475 |
| PCr Concentration | 2.1E-05 | 1.7E-05 | 1.56E-05 | 1.85E-05 |
| PCr Estimation Error (%SD) | 326.25 | 603.5455 | 604.5833 | 597.8333 |
| PCr Concentration Ratio to Total Ch | 1.49325 | 0.597364 | 1.005583 | 1.846083 |
| Scyllo Concentration | 2.43E-06 | 1.56E-06 | 1.1E-06 | 3.13E-06 |
| Scyllo Estimation Error (%SD) | 417 | 281.0909 | 433.25 | 188.25 |
| Scyllo Concentration Ratio to Total Ch | 0.110167 | 0.063864 | 0.062858 | 0.116583 |
| Tau Concentration | 9.46E-06 | 6.24E-06 | 2.31E-06 | 3.09E-06 |
| Tau Estimation Error (%SD) | 561.8333 | 580 | 748.75 | 427.1667 |
| Tau Concentration Ratio to Total Ch | 0.358333 | 0.231364 | 0.141917 | 0.277083 |
| **Hippocampus** | | | | |
| Ala Concentration | 2.31E-05 | 7.69E-06 | 6.6E-06 | 1.87E-05 |
| Ala Estimation Error (%SD) | 295.4167 | 540.0909 | 638.5 | 460 |
| Ala Concentration Ratio to Total Ch | 1.647833 | 0.340909 | 0.361833 | 1.150917 |
| Asp Concentration | 3.51E-06 | 6.04E-06 | 1.24E-05 | 7.49E-06 |
| Asp Estimation Error (%SD) | 492.6667 | 414.9091 | 313.1667 | 346 |
| Asp Concentration Ratio to Total Ch | 0.204658 | 0.295364 | 184.0231 | 0.384083 |
| Cr Concentration | 2.8E-05 | 2.85E-05 | 2.78E-05 | 3.47E-05 |
| Cr Estimation Error (%SD) | 177.4167 | 211.9091 | 269.8333 | 123.8333 |
| Cr Concentration Ratio to Total Ch | 1.61025 | 1.352909 | 1.811583 | 1.567583 |
| Cr+PCr Concentration | 4.93E-05 | 4.43E-05 | 3.9E-05 | 5.09E-05 |
| Cr+PCr Estimation Error (%SD) | 10.5 | 92.27273 | 14.83333 | 9 |
| Cr+PCr Concentration Ratio to Total Ch | 2.892667 | 2.107364 | 39.56108 | 2.673667 |
| CrCH2 Concentration | 7.59E-06 | 5.73E-06 | 3.09E-06 | 1.22E-05 |
| CrCH2 Estimation Error (%SD) | 487.0833 | 684.9091 | 626.8333 | 556.3333 |
| CrCH2 Concentration Ratio to Total Ch | 0.473508 | 0.239164 | 141.8788 | 0.544508 |
| GABA Concentration | 4.64E-06 | 5.09E-06 | 3.72E-06 | 5.64E-06 |
| GABA Estimation Error (%SD) | 145.1667 | 209.7273 | 314.1667 | 273 |
| GABA Concentration Ratio to Total Ch | 0.2515 | 0.246309 | 0.243833 | 0.283417 |
| GPC Concentration | 1.49E-05 | 1.31E-05 | 1.33E-05 | 1.78E-05 |
| GPC Estimation Error (%SD) | 181.3333 | 202.5455 | 188.6667 | 114.9167 |
| GPC Concentration Ratio to Total Ch | 0.795833 | 0.705636 | 0.85825 | 0.81275 |
| GPC+PCh Concentration | 1.8E-05 | 1.98E-05 | 1.51E-05 | 2.08E-05 |
| GPC+PCh Estimation Error (%SD) | 10.91667 | 11.36364 | 93.91667 | 8.666667 |
| GPC+PCh Concentration Ratio to Total Ch | 1 | 1 | 1 | 1 |
| GSH Concentration | 9.17E-06 | 1.63E-05 | 1.17E-05 | 1.12E-05 |
| GSH Estimation Error (%SD) | 205.5833 | 236.1818 | 63.41667 | 139.5833 |
| GSH Concentration Ratio to Total Ch | 0.580525 | 0.761091 | 53.08683 | 0.665917 |
| Glc Concentration | 5.59E-06 | 9.8E-06 | 4.86E-06 | 1.09E-05 |
| Glc Estimation Error (%SD) | 181.8333 | 160.6364 | 235.9167 | 52.58333 |
| Glc Concentration Ratio to Total Ch | 0.323333 | 0.497545 | 175.2729 | 0.5255 |
| Gln Concentration | 4.43E-05 | 4.06E-05 | 2.83E-05 | 3.02E-05 |
| Gln Estimation Error (%SD) | 38.83333 | 120.6364 | 74.08333 | 135.0833 |
| Gln Concentration Ratio to Total Ch | 2.482083 | 1.930545 | 177.0304 | 1.85125 |
| Glu Concentration | 5.39E-05 | 6.69E-05 | 4.95E-05 | 6.66E-05 |
| Glu Estimation Error (%SD) | 26.33333 | 106.5455 | 124.1667 | 20.08333 |
| Glu Concentration Ratio to Total Ch | 3.009917 | 3.191364 | 37.98142 | 3.4455 |
| Glu+Gln Concentration | 9.82E-05 | 0.000108 | 7.78E-05 | 9.68E-05 |
| Glu+Gln Estimation Error (%SD) | 16 | 101.3636 | 33.66667 | 14.91667 |
| Glu+Gln Concentration Ratio to Total Ch | 5.491917 | 5.122 | 213.1243 | 5.296833 |
| Ins Concentration | 6.36E-05 | 6.32E-05 | 5.64E-05 | 6.18E-05 |
| Ins Estimation Error (%SD) | 10.41667 | 10.54545 | 95.25 | 9.333333 |
| Ins Concentration Ratio to Total Ch | 3.835667 | 3.571727 | 220.8213 | 3.20475 |
| Lac Concentration | 4.55E-06 | 7.31E-06 | 1.12E-05 | 5.86E-06 |
| Lac Estimation Error (%SD) | 713.0833 | 427.8182 | 307.4167 | 571 |
| Lac Concentration Ratio to Total Ch | 0.303333 | 0.364909 | 25.05992 | 0.315583 |
| Lip09 Concentration | 3.11E-05 | 2.99E-06 | 1.07E-05 | 1.34E-05 |
| Lip09 Estimation Error (%SD) | 460.6667 | 704.9091 | 719.3333 | 643.5833 |
| Lip09 Concentration Ratio to Total Ch | 1.4827 | 0.133545 | 0.6275 | 0.738967 |
| Lip13a Concentration | 0.000179 | 1.97E-05 | 3.53E-05 | 7.65E-05 |
| Lip13a Estimation Error (%SD) | 272.0833 | 563.0909 | 400.1667 | 406.0833 |
| Lip13a Concentration Ratio to Total Ch | 11.37542 | 0.924909 | 2.276833 | 3.761667 |
| Lip13a+Lip13b Concentration | 0.00018 | 2.31E-05 | 3.99E-05 | 7.88E-05 |
| Lip13a+Lip13b Estimation Error (%SD) | 55.91667 | 346.4545 | 242.6667 | 270.0833 |
| Lip13a+Lip13b Concentration Ratio to Total Ch | 11.49292 | 1.375909 | 2.462 | 4.0155 |
| Lip13b Concentration | 1.8E-06 | 3.35E-06 | 4.57E-06 | 2.32E-06 |
| Lip13b Estimation Error (%SD) | 782.8333 | 753.1818 | 841.5 | 857.1667 |
| Lip13b Concentration Ratio to Total Ch | 0.1175 | 0.450936 | 0.185167 | 0.253833 |
| Lip20 Concentration | 1.48E-05 | 1.73E-06 | 3.57E-06 | 6.14E-06 |
| Lip20 Estimation Error (%SD) | 287.6667 | 651.3636 | 539.25 | 561.5 |
| Lip20 Concentration Ratio to Total Ch | 0.761167 | 0.076173 | 0.2155 | 0.319433 |
| MM09 Concentration | 4.73E-05 | 3.83E-05 | 7.76E-05 | 6.63E-05 |
| MM09 Estimation Error (%SD) | 186.3333 | 197.1818 | 100.5 | 19.75 |
| MM09 Concentration Ratio to Total Ch | 3.122083 | 1.813125 | 6.988333 | 3.389583 |
| MM09+Lip09 Concentration | 7.84E-05 | 4.13E-05 | 8.83E-05 | 7.97E-05 |
| MM09+Lip09 Estimation Error (%SD) | 17.33333 | 194.8182 | 98.75 | 15.25 |
| MM09+Lip09 Concentration Ratio to Total Ch | 4.604917 | 1.946671 | 7.615917 | 4.1285 |
| MM12 Concentration | 1.7E-05 | 1.26E-05 | 3.11E-05 | 2.64E-05 |
| MM12 Estimation Error (%SD) | 114.9167 | 219.1818 | 129.25 | 43.25 |
| MM12 Concentration Ratio to Total Ch | 1.117333 | 0.592386 | 2.538167 | 1.325083 |
| MM14 Concentration | 5.43E-05 | 3.6E-05 | 8.39E-05 | 6.03E-05 |
| MM14 Estimation Error (%SD) | 224.5 | 312.2727 | 115.25 | 140.5833 |
| MM14 Concentration Ratio to Total Ch | 3.19 | 1.762545 | 6.271 | 2.923833 |
| MM14+Lip13a+L Concentration | 0.000251 | 7.18E-05 | 0.000155 | 0.000165 |
| MM14+Lip13a+L Estimation Error (%SD) | 22.83333 | 45.63636 | 107.8333 | 33 |
| MM14+Lip13a+L Concentration Ratio to Total Ch | 15.80025 | 3.730727 | 11.27117 | 8.2645 |
| MM17 Concentration | 7.15E-05 | 3.85E-05 | 6.24E-05 | 7.42E-05 |
| MM17 Estimation Error (%SD) | 23 | 200.3636 | 103.9167 | 19.83333 |
| MM17 Concentration Ratio to Total Ch | 4.299167 | 1.830476 | 7.893667 | 3.69575 |
| MM20 Concentration | 6.77E-05 | 5E-05 | 8.29E-05 | 7.65E-05 |
| MM20 Estimation Error (%SD) | 196 | 294.4545 | 117.75 | 65.58333 |
| MM20 Concentration Ratio to Total Ch | 4.513833 | 2.331424 | 7.496583 | 4.4315 |
| MM20+Lip20 Concentration | 8.24E-05 | 5.17E-05 | 8.65E-05 | 8.26E-05 |
| MM20+Lip20 Estimation Error (%SD) | 34.16667 | 293.8182 | 116.75 | 38.91667 |
| MM20+Lip20 Concentration Ratio to Total Ch | 5.275083 | 2.407515 | 7.712167 | 4.750917 |
| NAA Concentration | 3.04E-05 | 4.03E-05 | 2.25E-05 | 4.71E-05 |
| NAA Estimation Error (%SD) | 163.1667 | 106.6364 | 206.5 | 16.25 |
| NAA Concentration Ratio to Total Ch | 1.653417 | 1.953182 | 31.52292 | 2.468667 |
| NAA+NAAG Concentration | 5.45E-05 | 5.75E-05 | 4.48E-05 | 6.71E-05 |
| NAA+NAAG Estimation Error (%SD) | 15.41667 | 99.54545 | 20.33333 | 10.58333 |
| NAA+NAAG Concentration Ratio to Total Ch | 3.05275 | 2.753545 | 33.16658 | 3.38025 |
| NAAG Concentration | 2.41E-05 | 1.73E-05 | 2.23E-05 | 1.99E-05 |
| NAAG Estimation Error (%SD) | 211.5833 | 214.5455 | 132.9167 | 141.4167 |
| NAAG Concentration Ratio to Total Ch | 1.3995 | 0.800273 | 1.643667 | 0.911667 |
| PCh Concentration | 3.1E-06 | 6.7E-06 | 1.82E-06 | 3.01E-06 |
| PCh Estimation Error (%SD) | 702.5833 | 568 | 839.5 | 762.1667 |
| PCh Concentration Ratio to Total Ch | 0.204167 | 0.294364 | 0.14175 | 0.18725 |
| PCr Concentration | 2.13E-05 | 1.58E-05 | 1.12E-05 | 1.63E-05 |
| PCr Estimation Error (%SD) | 294 | 486.1818 | 598.75 | 369.25 |
| PCr Concentration Ratio to Total Ch | 1.282417 | 0.754364 | 37.74958 | 1.106 |
| Scyllo Concentration | 6.93E-07 | 1.19E-06 | 1.25E-06 | 1.38E-06 |
| Scyllo Estimation Error (%SD) | 426.8333 | 296.9091 | 276.3333 | 431.75 |
| Scyllo Concentration Ratio to Total Ch | 0.040317 | 0.058182 | 43.07792 | 0.069775 |
| Tau Concentration | 1.66E-06 | 4.13E-06 | 4.54E-06 | 2.54E-06 |
| Tau Estimation Error (%SD) | 661.6667 | 530.6364 | 394.1667 | 599.25 |
| Tau Concentration Ratio to Total Ch | 0.11575 | 0.228273 | 200.3068 | 0.169485 |
